# Rapid wound healing in a reef manta ray masks the extent of vessel strike

**DOI:** 10.1371/journal.pone.0225681

**Published:** 2019-12-11

**Authors:** Frazer McGregor, Anthony J. Richardson, Amelia J. Armstrong, Asia O. Armstrong, Christine L. Dudgeon

**Affiliations:** 1 Murdoch University Field Station, 1 Banksia Drive Coral Bay, Australia; 2 Centre for Applications in Natural Resource Mathematics (CARM), School of Mathematics and Physics, The University of Queensland, QLD, Australia; 3 CSIRO Oceans and Atmosphere, Queensland Biosciences Precinct (QBP), QLD, Australia; 4 School of Biomedical Sciences, The University of Queensland, St. Lucia, Qld, Australia; Institut de recherche pour le developpement, FRANCE

## Abstract

Increasing vessel traffic in the marine environment due to commercial and recreational activities has amplified the number of conflicts with marine animals. However, there are limited multi-year observations of the healing rate of marine animals following vessel strike. Here we document the healing rate of a reef manta ray *Mobula alfredi*, following lacerations caused by a propeller along the pectoral fin. We demonstrate a high healing capacity, with wound length following a negative exponential curve over time. Lacerations healed to 5% of the initial wound length (i.e. 95% closure) within 295 days. The wounds appeared to stabilise at this point as observed more than three years following the incident and resulted in a distinctive scarring pattern. Examination of an extensive photo-identification catalogue of manta rays from the Ningaloo Coast World Heritage Area showed that the scarring pattern occurs more frequently than previously recognised, as the wounds had been previously attributed to failed predation attempts. This study provides baseline information for wound healing from vessel strike in reef manta rays and indirect evidence for increased vessel strikes on manta rays within the Ningaloo Coast World Heritage Area. We discuss the implication for spatial and behavioural management of vessels around manta rays.

## Introduction

Vessel traffic has increased substantially world-wide over the last three decades [1]. With increased traffic, more vessels strike marine fauna, which may have lethal or non-lethal outcomes [2]. Most information on vessel strikes concern marine mammals and reptiles [3–8] as they spend considerable time in surface waters to breathe, and float following lethal contact with a vessel [2, 3]. By contrast, impacts of vessel strikes on elasmobranchs has received little consideration.

Although elasmobranchs do not surface to breathe, several species spend considerable time in surface waters for activities such as basking and feeding, where they are more susceptible to vessel strike (e.g. basking shark *Cetorhinus maximus*, [9]; whale shark *Rhincodon typus*, [10]; reef manta ray *Mobula alfredi*, [11]; Chilean devil ray *Mobula tarapacana*, [12]; tiger shark *Galeocerdo cuvier*, [13]). Documenting vessel strike on elasmobranchs is challenging, as lethal impacts cause animals to sink [14] and non-lethal impacts may not be recognizable. Most wounds in elasmobranchs have been attributed to causes such as predation [15], mating attempts [16–18], and fishing related injuries or entanglement [19, 20]. Further, elasmobranchs are considered to have high healing capacity [18, 21], likely due in part to a unique adaptive immune system [22]. However, few studies have investigated the rate of wound healing in elasmobranchs, and these have focused on impacts of external and internal tagging procedures e.g. [18, 23]. Healing rates have been reported following predation attempts in reef manta rays [15, 24] and whale sharks [17]. There are only two examples of wound healing following vessel strike for elasmobranchs: a white shark *Carcharodon carcharias* [25] and a black tip reef shark *Carcharhinus melanopterus* [18]. However, documented recovery following vessel strike remains lacking for many species, including reef manta rays.

Here we report for the first time the rate of wound healing following vessel strike for a reef manta ray from the Ningaloo Coast World Heritage Area of Western Australia. Increased tourist visitation to this region to undertake in-water interactions with charismatic megafauna, as well as recreational fishing, has led to a simultaneous increase in vessel traffic [26, 27], a trend which is likely to continue. Based on the characteristic healing patterns, we identified the proportion of the manta ray population within the region that is likely to have been impacted by vessel collision. Implications for manta ray ecotourism here and in other regions are discussed.

## Methods

**Fig 1:**
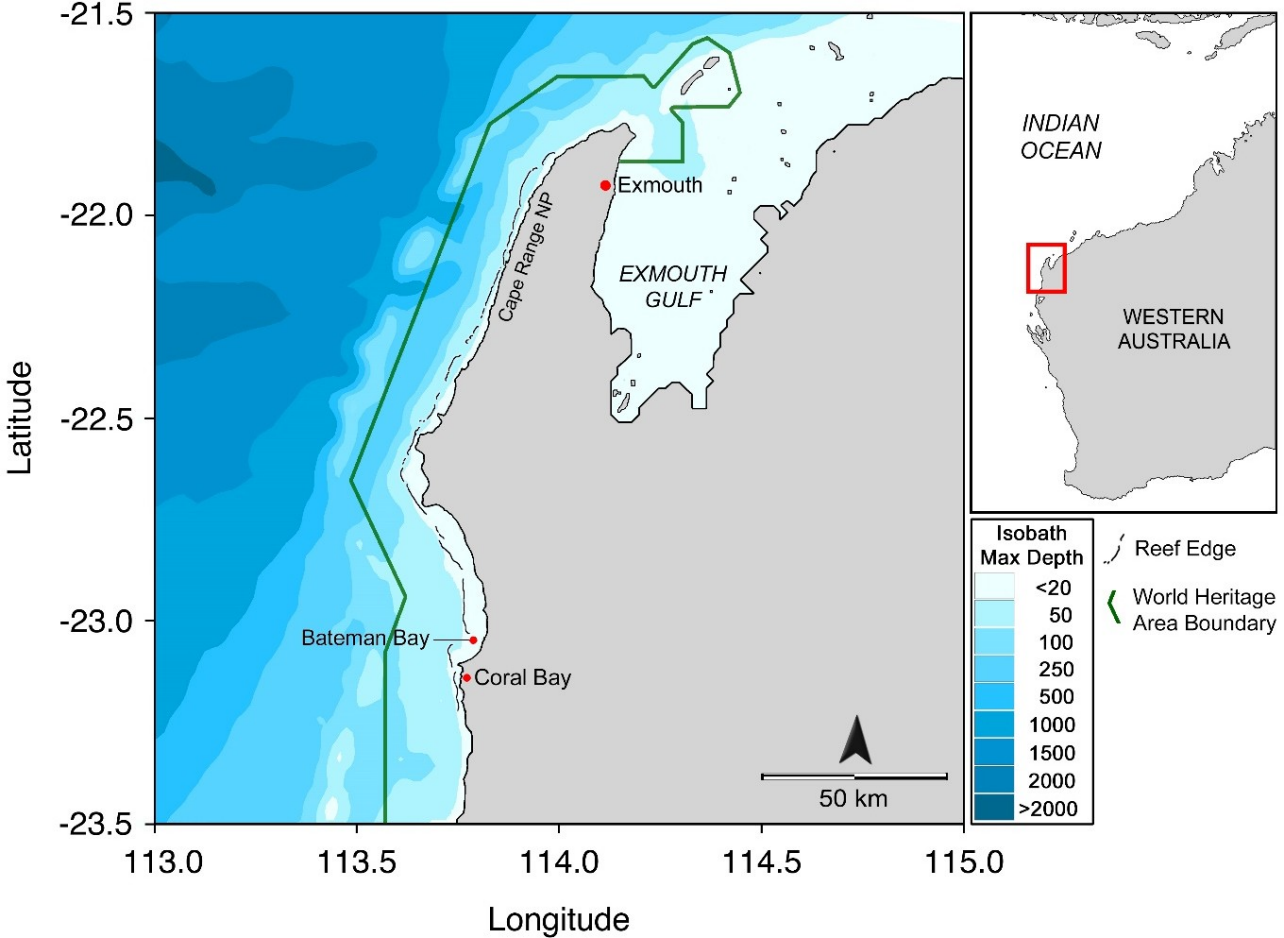
Map of the study area showing the outline of the Ningaloo Coast World Heritage Area in Western Australia, and key reef manta ray *Mobula alfredi* monitoring areas: Bateman Bay to the north of the Coral Bay township, waters to the western side of Cape Range National Park and the northern end of the Exmouth Gulf near Exmouth township. Figure created using the R package ‘marmap’ [44] with bathymetric data from the ETOPO1 database [45] and the marine component of protected area bounds from The World Database on Protected Areas [46]. Major reef features were added in Adobe Photoshop, from geographically aligned Landsat-8 imagery courtesy of the U.S. Geological Survey.

We conducted our study in the Ningaloo Coast World Heritage Area of Western Australia. The reef manta rays of this region have been the focus of a photographic-identification study spanning 15 years (2004–2019). Individuals are identified based on unique ventral patterning [24, 28]. Photographs of manta rays are obtained throughout the year from dedicated research trips, citizen science contributions, and from the tourism industry. Ancillary observations such as behaviour, maturity and obvious scarring are included in the database. Key areas for monitoring and observation of reef manta rays in the Ningaloo Coast World Heritage Area are: (1) All of Bateman Bay, containing several cleaning stations and important foraging grounds; (2) a 20km stretch of reef along the western side of Cape Range National Park between Tantabiddi boat ramp and Milyering; and (3) the western edge of Exmouth Gulf from the town marina to Bundegi (Fig 1). Within Bateman Bay monitoring occurs on a daily basis throughout the year, whilst the other locations have been largely opportunistic during Autumn and Spring respectively.

On the 30 June 2015, a female reef manta ray (catalogue number #0018) was observed at Point Maud in southern Bateman Bay (23^o^07.460’S, 113^o^45.630’E). Manta ray #0018 had noticeable recent wounds, comprising five full thickness cuts on the trailing edge of the left pectoral fin. Characteristics of the wounds were consistent with those inflicted by propeller strikes as identified on other marine megafauna [5, 17, 25, 29]. Manta ray #0018 is the focus of this study.

Manta ray #0018 is a melanistic morph reef manta ray *Mobula alfredi* with black dorsal and ventral surfaces and a unique white ventral pattern. Since the first sighting in 2004, manta ray #0018 has been observed displaying pre-mating behaviours as described in other manta ray populations [30], and subsequently observed heavily pregnant on several occasions, indicating sexual maturity. A small incision or wound on the right pectoral fin and a slightly crooked tail were documented during the earliest sighting of this individual, and both have remained unchanged through time. Measurements via laser photogrammetry were obtained in 2009 and again in 2018, with 3.69 m and 3.79 m disc widths recorded respectively [31]. Demonstrating seasonal visitation to Bateman Bay between April and August each year, behavioural observations associated with sighting records show manta ray #0018 primarily transiting the shallow surface waters (~2 m depth), visiting shallow (<10 m) cleaning stations, and surface feeding.

To assess wound healing rates and scarring characteristics, detailed images of manta ray #0018 were collected when the wounds were first observed and on each subsequent sighting (up until 24 July 2018). Images were collected using laser photogrammetry with laser pointers mounted 50 cm apart on either side of the camera (for detailed methodology see Deakos 2010). From the photographs, wound dimensions were estimated at each point in time using ImageJ software [32]. For a given photograph to be used for analysis it needed to be as close as possible to the same relative angle to the dorsal side of the target animal. Minor differences are acknowledged.

We analysed variation in healing rates among cut sites using mixed linear effects models implemented in the R package ‘lme4’ [33, 34]. Cut lengths were log transformed and the data set reduced to <300 days because the wound had virtually fully healed after this time. Healing rates among the five wound sites showed little variation and the inclusion of the cut site as a random variable was not significant. We fitted a negative exponential using a nonlinear least squares regression on pooled cut sites and using the R package ‘nls2’ [35]:

$$y=Ae^{-Bx}+C$$

Where *y* = cut length (y), and *A* = length of cuts at Day 0, *B* = exponential rate of decay, and *C* = estimates the length of healed cuts. As r^2^ (the proportion of variance explained) is not strictly valid for nls models, we calculated pseudo-r^2^ as the square of the correlation between observed and predicted values. The dataset is available for download through eSpace at the University of Queensland (doi.org/10.14264/uql.2019.869).

To assess the potential frequency of vessel strike on manta rays in the Ningaloo Coast World Heritage Area population, we interrogated the regional photo-identification database. Since the commencement of the database in 2004, metadata on scarring visible in images has been recorded, as well location on the animals and likely cause based on wound characteristics. Missing tissue (most commonly concave in shape) was assigned as predation attempts whilst slices or v-shaped cuts were assigned as anthropogenic (most likely propeller if along pectoral fins and entanglement or undetermined if on cephalic lobes). However, prior to the new information on the scarring patterns following the wound healing in manta ray #0018, similar scars may have been originally assigned to other threats such as predation attempts, rather than vessel strike, underestimating the occurrence of vessel-wildlife conflict. We compared wound classifications that had been conducted prior to the time of injury to manta ray #0018 in June 2015 (805 individuals), to a reassessment of all photographs in the database after healing had been completed in July 2018 (1071 individuals).

## Results

### Injury observations and rates of healing

**Fig 2:**
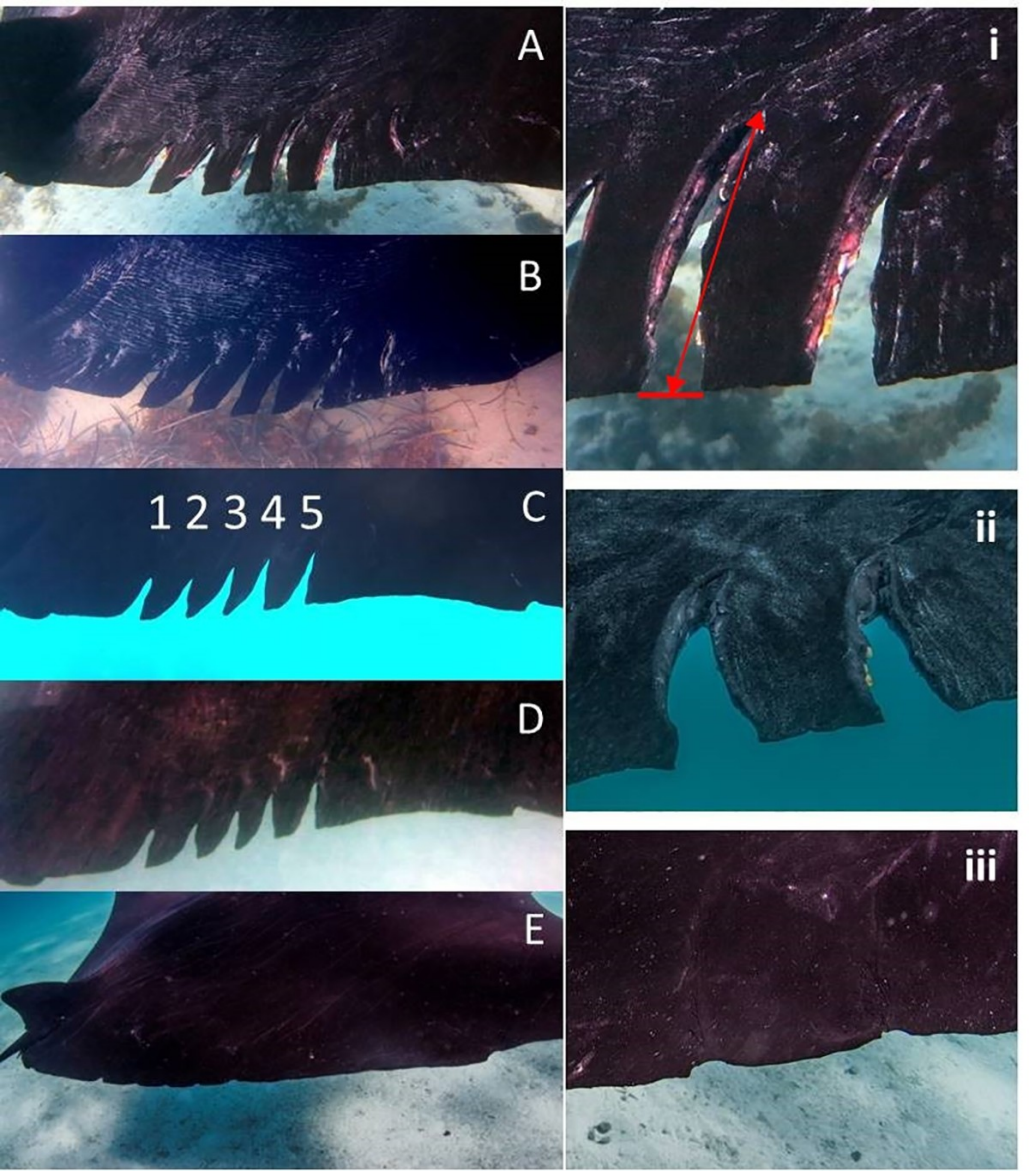
Images of the wounds from Days 0, 17, 33, 42 and 295 (A-E respectively) showing the fresh wound, incremental healing and near stable wounds on reef manta ray *Mobula alfredi* #0018. On the right are close up images of Day 0 (i) showing severed cartilage, Day 7 (ii) (time not used in calculations) showing the start of healing, and Day 295 (iii) showing near complete healing with concave scars. The red arrows in (i) indicate the protocol for measuring each wound where the distance from the top of the wound to the mid-point between the adjacent sides at the extremity was used to indicate wound length.

Changes in wound characteristics and stabilization (near complete healing) in manta ray #0018 was observed from photographs on seven separate occasions between 30 June 2015 and 24 July 2018 (Fig 2). When first observed (i.e. Day 0, 30 June 2015) there were five full thickness wounds ranging in length from 14.8–20.5 cm and a single surface wound (Fig 2A). Severed cartilage was visible in some wounds, as was red tissue indicative of recently stopped bleeding (haemostasis) (Fig 2i). An additional kink and bulge in the tail was also observed adding to the one present since the earliest sightings in 2004. These were not assessed for healing but were likely caused by the same vessel strike and appear to have remained unchanged during this study. A close up observation of wounds on Day 7 (6 July 2015), showed clear signs of healing, with all tissues a uniform grey. Inflammation was still noticeable around the wound margins (Fig 2ii). By Day 17 (16 July 2015), the wounds were 23% healed and ranged in length from 11.3–14.1 cm (Fig 2B), with a linear healing rate of ~0.24 cm day^-1^ since the wounds were first observed. By Day 33 (2 August 2015), wounds had healed 33.5% and had reduced in length to between 9.6 and 12.4 cm (Fig 2C). The linear healing rate had slowed to 0.11 cm/day. By Day 42 (11 August 2015), healing of the wounds had reached 37.4% and 9–12.1 cm in length (Fig 2D). Linear healing rates had continued to slow to 0.07 cm/day as the wound margins were drawn together by new tissue growth. In mid to late August 2015, manta ray #0018 is presumed to have left the area of Bateman Bay with no further sightings in 2015. When manta ray #0018 was next re-sighted on Day 295 (19 April 2016), measurements of the wounds showed they were 93.2% healed (Fig 2E), with a length ranging from 0.8–1.8 cm. Re-modelling of tissue appeared complete, with the wounds presenting as a series of small concave nicks along the trailing edge of the pectoral fin (Fig 2iii).

**Fig 3:**
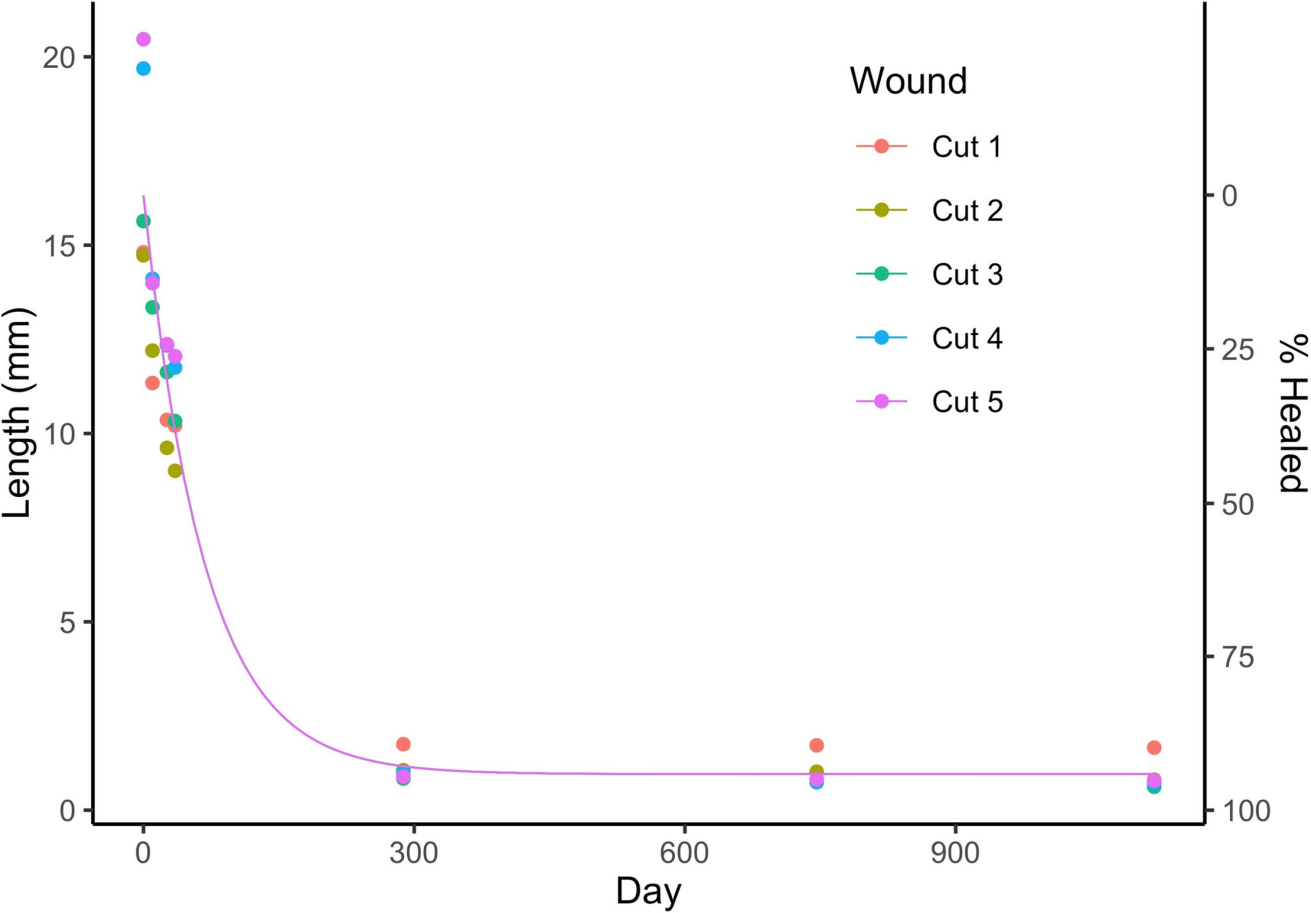
Wound healing rates from reef manta ray *Mobula alfredi* #0018. The wound length (mm) and percentage of wound healed over time, with Day 0 representing the first observation of the animal with fresh (severed cartilage and red tissue) wounds. A negative exponential model was fit to the data (y = 15.4e^-0.0149x^ + 0.956, pseudo-r^2^ = 0.96).

The average wound closure rate across the five wound sites was well fitted by a negative exponential model (y = 15.4e^-0.0149x^ + 0.956, pseudo-r^2^ = 0.96, Fig 3). From the model, the mean length of cuts at Time 0 is 15.4 cm. The asymptotic value of y for long time periods is 0.95, implying that cuts leave a 1 cm mark. The half-life for wound healing is -log(0.5)/-0.0149, which is 46.3 days. The model fit the data, with early healing resulting in 50% wound closure by Day 46. Wounds stabilized by Day 295 at 95% closed, between 0.6 and 1.7 cm in length (Fig 3).

### Origins of wounds

**Fig 4:**
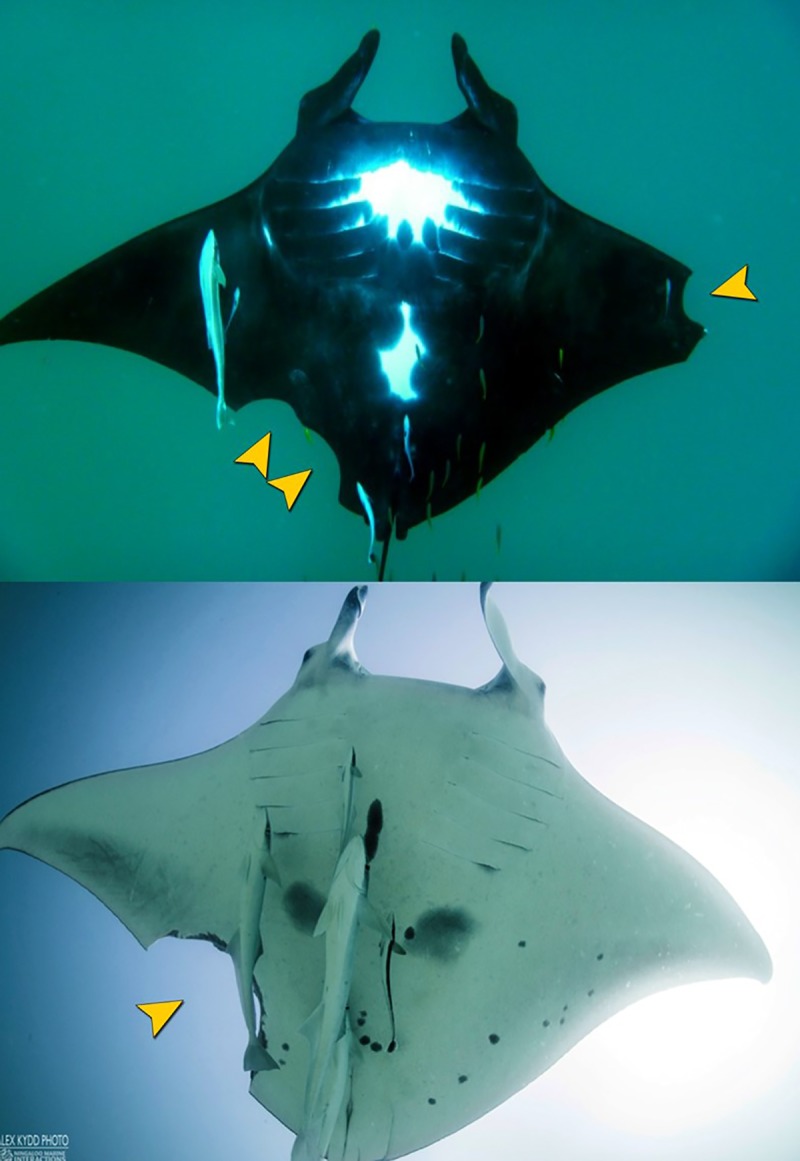
Examples of clear predation events on reef manta rays *Mobula alfredi*. Yellow arrows indicate the bite site on the fins.

As of July 2018, there were 1071 individuals documented in the Ningaloo Coast World Heritage area photo-identification database, with a total of 243 or 22.7% of individuals recorded with scars (Table 1). Twenty-eight individuals (2.6% of the total database) had large wounds with missing tissue on pectoral fins that were attributed unambiguously to predation attempts (Fig 4). Wounds and persistent scars with clear characteristics of propeller strike or other anthropogenic origins were present in 12 individuals (1.1% of the total database). All propeller strike observations were made in Bateman Bay and at no specific time of the year. Wounds that were observed on only the cephalic lobes or base of the tail in 30 animals, made it difficult to determine the cause, unless they were in early stages of healing. The largest scarring category included 140 individuals (13.1% of the total database) with minor concave nicks on the trailing edge of the pectoral fins. Scars with unknown cause represent a likely underestimation in wounds caused by boat strike as prior to the injury to manta ray #0018 the majority of these scars had been categorized as predation attempts (Table 1).

**Table 1:** The location and origin of scars on reef manta rays *Mobula alfredi* in the Ningaloo Coast World Heritage Area. Values are proportions of the images in the total photo identification database from the 1071 individual reef manta rays as of July 2018. Values in parentheses are database proportions (805 individuals) assigned prior to injury to manta ray #0018. Descriptions of wound types in parentheses refer to the scars with undetermined causes.

Origin of scar (description)	Location of scar				
	Cephalic Lobe	Base of tail	Dorsal Fin	Pectoral Fin	Totals
Predation Attempt	0. (0)	0.09(1.23)	0.09(0.12)	2.43(10.92)	**2.61(12.27)**
Propeller Injury	0. (0)	0.19(0)	0(0)	0.93(1.10)	**1.12(1.1)**
Undetermined Injury Totals	1.31(1.72)	1.21(0.37)	0.09(0.12)	16.34(0.25)	**18.95(2.45)**
(nicks)	0.93(1.23)	0(0)	0(0)	13.07(0)	**13.82(1.23)**
(slices)	0.28(0.37)	0.09(0)	0(0)	0.56(0)	**0.93(0.37)**
(missing tissue)	0.28(0.12)	1.12(0.37)	0.09(0.12)	2.52(0)	**4.01(0.61)**
(scratches)	0(0)	0(0)	0(0)	0.19(0.25)	**0.19(0.25)**
Totals	**1.31(1.72)**	**1.49(1.6)**	**0.19(0.25)**	**19.7(12.27)**	**22.69(15.83)**

## Discussion

Here we report for the first time on the rate of wound healing following vessel strike in a reef manta ray *Mobula alfredi*. We found initial rapid wound healing, with 37% of the wound closed after 33 days. The healing rate slowed over time following a negative exponential curve with 95% of the wound closed after 295 days. These findings are relevant to our understanding of wound healing processes in wild animals, specifically elasmobranchs, but also have significant implications for documenting vessel-wildlife conflict and implementing appropriate management regimes.

Our findings of rapid wound healing in a manta ray agrees with other studies on wound healing rates in elasmobranchs. For example, a large laceration (25 cm long, 30 cm wide, 8.5 cm deep) caused by a boat propeller in a wild juvenile white shark appeared to be completely healed nine months after the injury [25]. A study removing skin sections from a captive nurse shark *Ginglymostoma cirratum* and a leopard shark *Triakis semifasciata* found wounds to be completely covered with new denticles after four months, with differences in denticle formation between healed and non-impacted skin areas [36]. Even faster healing rates have been documented for wounds in wild blacktip reef sharks, including neonatal scars (scar area decreased by 94% in 24 days); bite wounds (20 cm wide wound completely closed in 40 days); presumed boat strike (24 cm wide wound 3–4 cm deep completely closed within 27 days) and tag insertion cut sites (healed within 29 days, no scar visible within 129 days) [18]. Shark bites on manta rays were observed to have completely healed within 126 to 225 days [15], with estimated healing rates likely conservative given the undocumented time point when healing was complete. Similarly, the reported wound healing in manta ray #0018 might have been faster if we were able to observe her more regularly between days 42 and 295.

Intrinsically, healing rates will be based on the physiology and immunology of the organism, but other factors may also have influence including type and severity of the wound, the wound location on the animal and extrinsic factors. For example, empirical studies on teleosts have shown faster generation of new tissue in warmer versus cooler waters [37]. Observations on wild elasmobranchs lend support to this idea, with slower healing rates of small abrasions and cuts in white sharks in colder waters [21] compared with sicklefin lemon sharks *Negaprion acutidens* [38] and blacktip reef sharks in tropical waters [18]. All observations for manta ray #0018 were from Bateman Bay, which is towards the latitudinal extent of the tropics. During the first couple of months following the injury the water temperature in the region ranged between 21–24°C. Her movements outside of this region are unknown, although reef manta rays tagged with satellite transmitters in Bateman Bay have moved approximately 450 km into cooler subtropical waters (Armstrong et al. 2019 submitted). Similar incursions into cooler subtropical waters have been demonstrated by reef manta rays at comparable latitudes in east Australia [39]. Therefore, even more rapid wound healing may occur in reef manta rays inhabiting tropical waters. Marine cleaner fish have been proposed to assist with wound healing in hosts through removal of injured or necrotic tissue [40]. There are several cleaning stations within Bateman Bay and manta ray #0018 is frequently observed visiting these stations, which may also promote wound healing.

By observing the wound healing through time we were able to determine the resulting scarring pattern. In the earlier assessment of the Ningaloo Coast World Heritage Area population, similar scarring patterns were designated as having predatory origins with the jagged wing tip features being attributed to teeth rakes from failed shark bites. Furthermore, studies of wound healing in manta rays from other populations have mainly reported on predation events and/or human impacts from fishing gear interactions, and these impacts differ across populations. For example, 76% of reef manta rays in a Mozambican population showed evidence of shark bites [15], with 96% of these injuries along the trailing edge of the pectoral fins. By contrast, just 24% of the Hawaiian population of reef manta rays exhibited shark bite injuries [20], while 10% of this same population from Hawaii displayed cephalic lobe injuries indicative of fishing line entanglement. In our reassessment of the Ningaloo population, only 29 individuals (2.7% of the population) had wounds that could be attributed unambiguously to predation events. However, 13.1% of the population showed scarring patterns similar to the healed wounds of manta ray #0018, suggesting that the incident of vessel strike on reef manta rays in this region may be substantially higher than previously identified.

The rapid and near complete wound healing and characteristic scarring pattern we describe in this study has implications for the management of reef manta rays both within the Ningaloo Coast World Heritage Area and elsewhere. Whilst high wound healing capacity is likely to be beneficial for the long-term survival of these animals, the fitness cost of injuries may be masked. It is unknown what energy reserves are required for wound healing and potential reductions in reproductive output and growth over the healing period. Further, the occurrence of vessel strike is likely to be underestimated, which may have economic as well as conservation implications. Manta rays are the focus of ecotourism activities world-wide, with estimates exceeding USD$140 million globally [41] and USD$1.2 million in the Ningaloo Reef Marine World Heritage area (McGregor unpublished data). Much of this activity is surface viewing from vessels or in-water surface interactions, with the local industry expanding from just one manta tourism vessel in the 1990s to present day with five dedicated manta tourism vessels [42]. Understanding the risk of vessel strike is important for management planning. Future research should focus on: (1) identifying priority habitat areas, (2) the travel corridors between these areas and the open water; and (3) investigating the spatial and temporal overlap of usage by wildlife and vessels [43]. This information in turn could be used to inform policy for designing adequate spatial management for this region to reduce reef manta ray-vessel impact and protect critical habitat. Such management may include dynamic zoning with speed restrictions during high usage periods, vessel free zones, the use of propeller guards or alternative motors (e.g. jet motors), and an increase in education to all stakeholders on vessel-wildlife conflict.
